# *In silico* characterization of chromosomally integrated bla_CTX-M_ genes among clinical *Enterobacteriaceae* in Africa: insights from whole-genome analysis

**DOI:** 10.3389/fmicb.2025.1655907

**Published:** 2025-09-12

**Authors:** Misheck Shawa, Herman Chambaro, Harvey K. Kamboyi, Clement Sulwe, Joseph Y. Chizimu, Situmbeko J. Nasilele, Shohei Ogata, Mulemba Samutela, Tuvshinzaya Zorigt, Steward Mudenda, Manyando Simbotwe, Mwamba Nsofwa, Jedidiah Chanda, Freeman Chabala, Mike Nundwe, Joseph Ndebe, Msangwa Sinjani, Kyoko Hayashida, Naganori Nao, Roma Chilengi, Hirofumi Sawa, Yasuhiko Suzuki, Bernard Hang'ombe, Masahiro Kajihara, Hideaki Higashi

**Affiliations:** ^1^Hokudai Center for Zoonosis Control in Zambia, Hokkaido University, Lusaka, Zambia; ^2^Division of International Research Promotion, International Institute for Zoonosis Control, Hokkaido University, Sapporo, Japan; ^3^Department of Pathobiology, College of Veterinary Medicine, University of Illinois Urbana-Champaign, Champaign, IL, United States; ^4^Department of Clinical Medicine, School of Medicine, Eden University, Lusaka, Zambia; ^5^Institute of Biochemistry, Molecular Biology and Biotechnology, University of Colombo, Colombo, Sri Lanka; ^6^Zambia National Public Health Institute, Ministry of Health, Lusaka, Zambia; ^7^Department of Biomedical Sciences, School of Veterinary Medicine, University of Zambia, Lusaka, Zambia; ^8^Department of Biomedical Sciences, School of Health Sciences, University of Zambia, Lusaka, Zambia; ^9^Division of Infection and Immunity, International Institute for Zoonosis Control, Hokkaido University, Sapporo, Japan; ^10^Department of Disease Control, School of Veterinary Medicine, University of Zambia, Lusaka, Zambia; ^11^Institute of Basic and Biomedical Sciences, Levy Mwanawasa Medical University, Lusaka, Zambia; ^12^Division of Collaboration and Education, International Institute for Zoonosis Control, Hokkaido University, Sapporo, Japan; ^13^One Health Research Center, Hokkaido University, Sapporo, Japan; ^14^Hokkaido University, Institute for Vaccine Research and Development, Sapporo, Japan; ^15^Division of Molecular Pathobiology, International Institute for Zoonosis Control, Hokkaido University, Sapporo, Japan; ^16^Africa Centre of Excellence for Infectious Diseases of Humans and Animals, School of Veterinary Medicine, University of Zambia, Lusaka, Zambia; ^17^Global Virus Network, Baltimore, MD, United States; ^18^Division of Bioresources, International Institute for Zoonosis Control, Hokkaido University, Sapporo, Japan; ^19^Department of Research and Innovation, Copperbelt University, Kitwe, Zambia

**Keywords:** *bla*_CTX-M_, *Enterobacteriaceae*, Africa, chromosomal, IS*Ecp1*, IS26

## Abstract

Antimicrobial resistance (AMR) mediated by extended-spectrum *β*-lactamases (ESBLs) is a growing global concern, particularly among *Enterobacteriaceae*. The CTX-M-type ESBLs, encoded by the *bla*_CTX-M_ gene, are of significant public health importance due to their high prevalence and broad geographic distribution. Typically located on plasmids and often co-occurring with other AMR genes, *bla*_CTX-M_ contributes to multidrug resistance (MDR). However, increasing evidence suggests secondary chromosomal integration of *bla*_CTX-M_, sometimes alongside other resistance determinants. The extent and implications of this mechanism remain poorly characterized, especially in Africa, where genomic surveillance is limited. In this study, we retrieved 295 chromosomal sequences of *Enterobacteriaceae* of African origin from the GenBank and performed *in silico* predictions of *bla*_CTX-M_ and other AMR genes. *bla*_CTX-M_-carrying sequences were further characterized by *in silico* multilocus sequence typing and genome annotation. Chromosomal insertions were identified through alignment with reference genomes. Overall, 47 of 295 sequences (15.9%) harbored the *bla*_CTX-M_ gene, with the highest prevalence in *Klebsiella pneumoniae* (29/157, 18.5%), followed by *Escherichia coli* (13/72, 18.1%), *Enterobacter* spp. (4/38, 10.5%), and *Shigella* spp. (1/12, 8.3%). The most common allele was *bla*_CTX-M-15_ (31/47, 66.0%), followed by *bla*_CTX-M-14_ (12/47, 25.5%), *bla*_CTX-M-55_ (3/47, 6.4%), and *bla*_CTX-M-27_ (1/27, 3.7%). Co-occurrence of *bla*_CTX-M_ with additional AMR genes was frequently observed, with integration events often associated with mobile genetic elements such as IS*Ecp1* and IS26. Notably, strains from the same hospital setting were phylogenetically related and shared sequence types and AMR gene profiles, suggesting local clonal dissemination. These findings reveal a notable presence of chromosomally integrated *bla*_CTX-M_ among African *Enterobacteriaceae*, frequently in association with other resistance genes, thereby facilitating stable MDR propagation independent of plasmid maintenance. This evolutionary adaptation may have significant implications for the persistence and spread of MDR in clinical settings.

## Introduction

Despite significant strides in developing novel antimicrobials, the treatment of bacterial infections has become increasingly challenging due to the widespread emergence of multidrug resistance (MDR), particularly among *Enterobacteriaceae*. MDR pathogens represent a clear and present danger that affects every populated continent of the globe (Bassetti et al., 2015). For instance, MDR bacteria are estimated to contribute to 45% of deaths in Africa (WHO, 2014), and in 2019 alone, MDR *Escherichia coli* and *Klebsiella pneumoniae* were associated with over 100,000 deaths globally (Murray et al., 2022). In *Enterobacteriaceae*, MDR is often mediated by extended-spectrum *β*-lactamases (ESBLs) (Zhang et al., 2015; Beyene et al., 2024). While TEM and SHV variants were historically predominant, the CTX-M-type ESBLs, which hydrolyze third-generation cephalosporins like cefotaxime, have now become the most prevalent globally (Castanheira et al., 2021). CTX-M type ESBLs are classified into five groups, CTX-M-1, CTX-M-2, CTX-M-8, CTX-M-9, and CTX-M-25, with the CTX-M-1 group being the most predominant. Recent meta-analyses in sub-Saharan Africa confirm CTX-M-15 (CTX-M-1 group) as the most reported ESBL type across human and animal health sectors (Olaitan et al., 2025). The pandemic clone *E. coli* ST131 producing CTX-M-15 type ESBL remains a key driver in Africa, mirroring global trends (Nicolas-Chanoine et al., 2014). The *bla*_CTX-M_ gene, which encodes CTX-M types ESBLs, is typically located on mobile genetic vectors called plasmids that often harbor multiple antimicrobial resistance (AMR) genes (Mikhayel et al., 2024). This facilitates the rapid dissemination of MDR phenotypes.

Although plasmids contribute significantly to AMR gene spread, they may impose a fitness cost on the bacteria due to the energetic burden of their replication and maintenance, thus increasing the chances of instability or loss (Carroll and Wong, 2018). Additionally, plasmids are susceptible to loss during cell division through segregational instability. To overcome these limitations and ensure stable inheritance of critical traits, plasmid-encoded genes, including *bla*_CTX-M_, may integrate into the bacterial chromosome, provided such insertions do not truncate essential genes or alter vital regulatory pathways (San Millan et al., 2015). Chromosomal integration of *bla*_CTX-M_ is frequently mediated by mobile elements such as IS*Ecp1* and IS26 (Gomi et al., 2022; Komori et al., 2024). Most studies have reported chromosomal *bla*_CTX-M_ gene insertions as small segments (<5,000 bp) containing few or no additional resistance genes (Mshana et al., 2015). However, emerging evidence suggests that larger chromosomal islands harboring *bla*_CTX-M_ alongside multiple AMR genes [e.g., *aac(3)-IIa, qnrB1*, *aac(6′)-Ib-cr5, bla*_OXA-1_, *dfrA14*, *catB3*, *tet(A)*] also exist. These *bla*_CTX-M_-carrying islands are usually mobilized by transposable elements, which facilitate their insertion at various chromosomal sites. Notably, Yoon et al. (2020) described *bla*_CTX-M_-carrying chromosomal segments in *K. pneumoniae*, while Goswami et al. (2020) reported similar findings in *E. coli*.

Previously, our research group reported large MDR chromosomal islands carrying *bla*_CTX-M_ in *E. coli* and *Enterobacter cloacae* from Zambia (Shawa et al., 2021; Shawa et al., 2025). However, the overall prevalence and genetic landscape of chromosomal *bla*_CTX-M_ across the African continent remain poorly characterized. With the growing availability of whole genome sequencing (WGS) data from African countries submitted to the National Center for Biotechnology Information (NCBI), this study used *in silico* analysis to estimate the prevalence of chromosomally integrated *bla*_CTX-M_ among *Enterobacteriaceae* of African origin and characterize the genetic context of these chromosomal insertions.

## Methodology

In September 2024, whole genome sequences of *Enterobacteriaceae* chromosomes (*E. coli*, *K. pneumoniae*, *Enterobacter* spp., *Salmonella* spp., and *Shigella* spp.) from African clinical sources were downloaded from the NCBI nucleotide database. To this end, we explored the NCBI using search terms including the bacterial species, host species, and country (e.g., “*Escherichia coli*, *Homo sapiens*, Kenya”) and filtered the results using a customized sequence length range of 1,000,000 to 6,000,000 bp. Fasta files of the output sequences were collected as a single folder for each country through a bulk download. The number of unique sequences per dataset was determined using Seqkit (rmdup function) (Shen et al., 2016).

*In silico* prediction of AMR genes was performed by ResFinder (Florensa et al., 2022), and *bla*_CTX-M_-harboring genomes were annotated using dfast version 1.3.2 (Tanizawa et al., 2018). The chromosome sequences were also subjected to *in silico* multilocus sequence typing (MLST) using the mlst database,^1^ which partly uses the PubMLST database^2^ (Jolley and Maiden, 2010). Chromosomal insertions harboring the *bla*_CTX-M_ gene were detected by aligning the annotated files to appropriate reference genomes using Mauve (Darling et al., 2004), and the results were visualized in genoPlotR version 0.8.11 (Guy et al., 2010). To further characterize the insertions, nucleotide sequences were subjected to BLASTn against the NCBI database, and the resulting hit tables were filtered in R using the dplyr package version 1.1.4 (Wickham et al., 2023), with filtering criteria set to “% identity > 99.” Additionally, plasmid replicons were detected using the PlasmidFinder database (Carattoli et al., 2014), while genomic islands of horizontal origin were predicted using IslandViewer 4 (Bertelli et al., 2017), which includes IslandPath-DIMOB (Hsiao et al., 2003), SIGI-HMM (Waack et al., 2006), and IslandPick (Langille et al., 2008). Previously published Zambian sequences were excluded from this analysis, as they have been characterized in prior studies (Shawa et al., 2021).

To distinguish between *E. coli* and *Shigella* spp., one sequence was subjected to a local BLAST using *lacY* and *ipaH* genes. To this end, *lacY* (from *E. coli* str. K-12 substr. MG1655, Accession Number: NC_000913.3) and *ipaH* (from *S. flexneri* 2a str. 301, Accession Number: NC_004337.2) were downloaded from the NCBI and concatenated into one fasta file. A local database was then created from the merged fasta file using the “makeblastdb” command while specifying the “-dbtype nucl” and “-parse_seqids” arguments. Finally, the query sequence was subjected to a BLAST search against the local database using the “blastn” command. *S. flexneri* 2a str. 2,457 T (Accession Number: AE014073.1) was used as a positive control for *ipaH*.

Core-SNP-based phylogenetic trees for the *bla*_CTX-M_-carrying genome sequences of each species were created using parsnp version 2.1.4 (Treangen et al., 2014) using *E. fergusonii* (Accession Number NZ_CP083638.1) and *K. quasipneumoniae* (Accession Number NZ_LR588411.1) as outgroups for *E. coli* and *K. pneumoniae*, respectively. Finally, the output parsnp tree files were visualized and edited in iTOL version 7 (Letunic and Bork, 2024).

## Results

### Chromosomal *bla*_CTX-M_ detected in ~ 16% of *Enterobacteriaceae* genomes

A total of 295 *Enterobacteriaceae* chromosomal sequences, each exceeding 1,000,000 bp, were retrieved from the NCBI nucleotide database (Table 1). The sequences originated from clinical isolates in 18 African countries and represented five genera: *K. pneumoniae* (*n* = 157), *E. coli* (*n* = 72), *Enterobacter* (*n* = 38), *Salmonella* (*n* = 16), and *Shigella* (*n* = 12) (Table 1). The genome sizes ranged from 1,000,114 to 5,772,140 bp. Out of 295 genomes analyzed, 47 (47/295, 15.9%) harbored the *bla*_CTX-M_ gene. The highest prevalence was observed in *K. pneumoniae* (29/157, 18.5%), followed by *E. coli* (13/72, 18.1%), *Enterobacter* (4/38, 10.5%), and *Shigella* (1/12, 8.3%) (Table 1). Geographically, chromosomal *bla*_CTX-M_ was detected in nine out of 18 countries (50%). The country-specific prevalence was; Sudan 94.4% (17/18), Ethiopia 20.9% (9/43), South Africa 6.0% (5/83), Uganda 40% (4/10), Ghana 42.9% (3/7), Nigeria 6.5% (3/46), Tanzania 14.3% (2/14), Malawi 25.0% (2/8), Niger 50% (1/2), and Egypt 5.6% (1/18) (Table 1). Among the *bla*_CTX-M_-carrying sequences, the most frequently detected allele was *bla*_CTX-M-15_ (31/47, 66.0%), followed by *bla*_CTX-M-14_ (12/47, 25.5%), *bla*_CTX-M-55_ (3/47, 6.4%), and *bla*_CTX-M-27_ (1/27, 3.7%). Six out of 29 *K. pneumoniae* sequences possessed two copies of *bla*_CTX-M_, while other species had a single copy of the gene.

**Table 1:** Distribution of chromosomal *Enterobacteriaceae* sequences downloaded from the GenBank.

Species	Country	No. of sequences downloaded	No. of sequences with chromosomal *bla*_CTX-M_
*Enterobacter*	Egypt	2	1
	Ghana	1	1
	Kenya	1	0
	Nigeria	22	2
	Senegal	1	0
	South Africa	11	0
	Total	38	4 (10.5%)
*E. coli*	Botswana	1	0
	Egypt	6	0
	Ethiopia	39	9
	Guinea	2	0
	Libya	1	0
	Malawi	6	2
	Mozambique	1	0
	Niger	2	1
	Nigeria	3	0
	Somalia	5	0
	South Africa	3	1
	Tanzania	3	0
	Total	72	13 (18.1%)
*K. pneumoniae*	Botswana	1	0
	Egypt	10	0
	Ethiopia	4	0
	Ghana	6	2
	Kenya	6	0
	Libya	7	0
	Malawi	2	0
	Nigeria	11	0
	Senegal	2	0
	South Africa	69	4
	Sudan	18	17
	Tanzania	11	2
	Uganda	10	4
	Total	157	29 (18.5%)
*Shigella*	Nigeria	10	1
	Somalia	2	0
	Total	12	1 (8.3%)
*Salmonella*	Kenya	13	0
	Senegal	2	0
	Tunisia	1	0
	Total	16	0 (0%)
Overall total		295	47 (15.9%)

Of the 47 *bla*_CTX-M_-harboring sequences, 45 were larger than 4.6 Mbp, while two *Enterobacter* sequences were 1,274,920 bp and 1,023,858 bp (Supplementary Table S1). Interestingly, plasmid replicons were detected in some chromosomes of *bla*_CTX-M_-carrying *K. pneumoniae* (19/29, 65.5%) and *E. coli* (2/13, 15.4%), all of which were larger than 4.9 Mbp (Table 2). The replicons included *p0111*, *IncFIB(AP001918)*, *IncFII, Col440I, Col(BS512)*, *ColpVC*, *IncR*, *IncFIB(pKPHS1)*, *IncA/C2*, *IncFII_1_pKP91*, and *IncFIB(K)_1_Kpn3* (Table 2).

**Table 2:** Plasmid replicons detected among chromosomal sequences.

ID	Accession #	Species	Contig size (bp)	Replicons
131	CP093011.1	*E. coli*	5,219,097	*p0111, IncFIB(AP001918), IncFII, Col440I, Col(BS512), ColpVC*
Past_Mal_6	NZ_JBFOBQ010000001.1	*E. coli*	4,901,674	*IncFIB(AP001918)*
28spn	CP092527.1	*K. pneumoniae*	5,694,380	*IncR* *IncFIB(pKPHS1), IncA/C2*
56spn	CP092528.1	*K. pneumoniae*	5,585,531	*IncFIB(pKPHS1), IncA/C2*
12spc	CP092917.1	*K. pneumoniae*	5,772,140	*IncR* *IncFIB(pKPHS1), IncA/C2*
MAKM-RS081	CP129536.1	*K. pneumoniae*	5,319,428	*IncFII_1_pKP91*
MAKM-3381	CP129541.1	*K. pneumoniae*	5,321,788	*IncFIB(K)_1_Kpn3*
S-P-N-044.01	CP092693.1	*K. pneumoniae*	5,581,521	*IncR, IncFIB(pKPHS1)_1_pKPHS1*
S-P-N-036.01	CP092695.1	*K. pneumoniae*	5,678,493	*IncR, IncFIB(pKPHS1)_1_pKPHS1, IncA/C2*
S-P-N-042.01	CP092696.1	*K. pneumoniae*	5,688,292	*IncR, IncFIB(pKPHS1)_1_pKPHS1, IncA/C2, Col156*
S-P-N-043.0	CP092697.1	*K. pneumoniae*	5,674,093	*IncR, IncFIB(pKPHS1)_1_pKPHS1, IncA/C2, Col440I, Col(KPHS6)*
S-P-C-013.01	CP092805.1	*K. pneumoniae*	5,736,610	*IncR, IncFIB(pKPHS1)_1_pKPHS1,* *IncA/C2*
S-P-C-007.01	CP092806.1	*K. pneumoniae*	5,757,953	*IncR, IncFIB(pKPHS1)_1_pKPHS1, IncA/C2*
S-P-C-024.01	CP092807.1	*K. pneumoniae*	5,675,175	*IncR, IncFIB(pKPHS1)_1_pKPHS1, IncA/C2*
S-P-C-028.01	CP092808.1	*K. pneumoniae*	5,738,885	*IncR, IncFIB(pKPHS1)_1_pKPHS1, IncA/C2*
S-P-N-031.01	CP092809.1	*K. pneumoniae*	5,697,977	*IncR, IncFIB(pKPHS1)_1_pKPHS1, IncA/C2, Col440I, Col156*
S-P-C-027.01	CP092810.1	*K. pneumoniae*	5,757,041	*IncR, IncFIB(pKPHS1)_1_pKPHS1, IncA/C2*
S-P-C-032.01	CP092811.1	*K. pneumoniae*	5,703,728	*IncR, IncFIB(pKPHS1)_1_pKPHS1, IncA/C2*
S-P-C-037.01	CP092812.1	*K. pneumoniae*	5,672,868	*IncR, IncFIB(pKPHS1)_1_pKPHS1, IncA/C2*
S-P-C-016.01	CP092813.1	*K. pneumoniae*	5,702,698	*IncR, IncA/C2*
S-P-N-054.01	CP092840.1	*K. pneumoniae*	5,770,326	*IncR, IncFIB(pKPHS1)_1_pKPHS1, IncA/C2, Col156*

### Multidrug-resistant chromosomal insertion (~12.8 kbp) identified in a Malawian *E. coli* sequence

**Figure 1:**
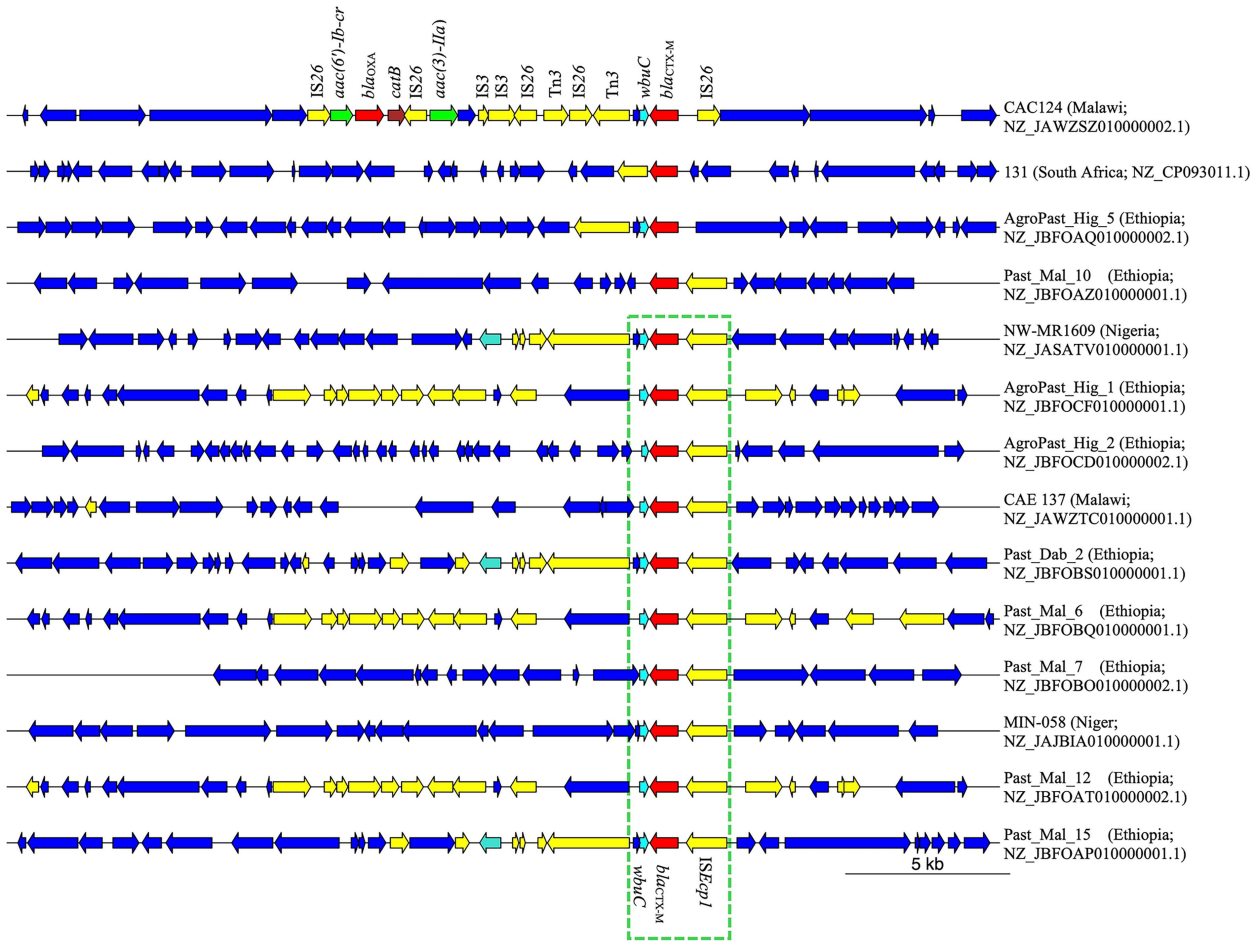
Genetic environment of *bla*_CTX-M_ in *Shigella* and *E. coli* chromosomes from Africa. From a total of 14 genomes, the IS*Ecp1* was observed upstream of *bla*_CTX-M_ in 11 strains, while *wbuC* existed downstream of *bla*_CTX-M_ in strains. CAC124 from Malawi exhibited multiple AMR genes on a composite transposon bracketed by directly oriented IS26 elements. Yellow; mobile genetic elements. Red; *β*-lactamase gene. Cyan; *wbuC*. Green; aminoglycoside resistance gene. Brown; chloramphenicol resistance gene.

**Figure 2:**
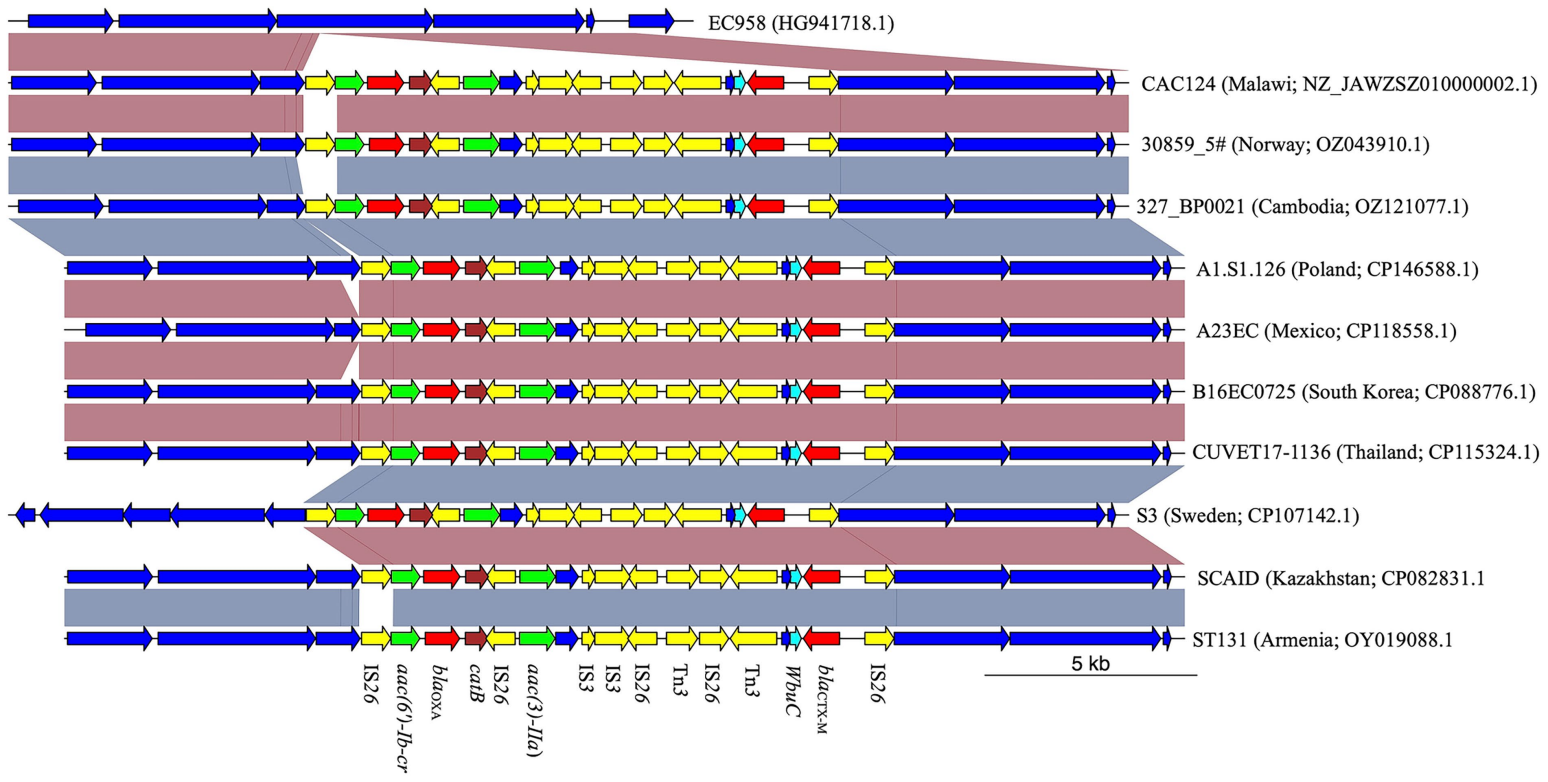
Comparison analysis of CAC124 from Malawi with *E. coli* ST131 strains from various countries across the world. The IS26-flanked composite transposon in CAC124 was found in several *E. coli* ST131. Yellow; mobile genetic elements. Red; β-lactamase gene. Cyan; *wbuC*. Green; aminoglycoside resistance gene. Brown; chloramphenicol resistance gene.

Among the 13 *E. coli* genomes analyzed, nine distinct sequence types (STs) were identified, with ST38 and ST450 observed twice. The remaining STs were unique to individual strains (Supplementary Table S2). Two isolates could not be assigned to an ST due to uncertainties in the *adk* allele. Notably, strain NW-MR1609 (Accession Number NZ_JASATV010000001.1), previously identified as *S. sonei*, was reassigned to *E. coli* ST484. This strain possessed *lacY*, an *E. coli* hallmark gene (Horakova et al., 2008), but lacked *ipaH*, which is present in all *Shigella* (Ashida and Sasakawa, 2015). Analysis of genomic regions flanking the *bla*_CTX-M_ gene revealed the presence of IS*Ecp1* insertion sequence 255 bp upstream in all but three strains. Furthermore, a 273 bp gene encoding the WbuC family cupin fold metalloprotein was located 46 bp downstream of the *bla*_CTX-M_ gene in all but two strains (Figure 1). Four strains exhibited additional AMR genes within 10,000 bp of *bla*_CTX-M_. Strains NW-MR1609 (ST484, Nigeria), Past_Dab_2 (ST8130, Ethiopia), and Past_Mal_15 (ST38, Ethiopia) each carried the *qnrS1* gene 4,640 bp downstream of *bla*_CTX-M_. The *qnrS1* gene was part of a 5,250 bp genomic island that was immediately adjacent to *bla*_CTX-M_ and harbored the Tn3-like element Tn3 family transposase gene (Supplementary Figure S1). The CAC124 strain from Malawi (ST5640, Accession Number NZ_JAWZSZ010000002.1) harbored *bla*_OXA-1_, two aminoglycoside-encoding genes [*aac(6′)-Ib-cr* and *aac(3)-IIa*], and *catB* for chloramphenicol resistance, all located within a 12,837 bp chromosomal insertion that lacked IS*Ecp1* (Figure 1). Compared to the reference strain (EC958, Accession Number HG941718.1), the CAC124 insertion was a composite transposon flanked by directly oriented IS26 elements, but the insertion point lacked target-site duplications (TSDs). Additional interspersed IS26 copies were observed adjacent to AMR genes or other transposable elements (Figure 1). A BLAST search revealed that this insertion was present in multiple sequences, including chromosomes from clinical *E. coli* ST131 strains isolated in over 30 countries worldwide (Supplementary Table S3; Figure 2). Furthermore, this sequence was part of a 13,577 bp genomic island bounded by hypothetical protein-encoding genes immediately external to the insertion’s flanking IS26 elements (Supplementary Figure S2).

### MDR chromosomal insertions identified in two *E. cloacae* strains from West Africa

**Figure 3:**
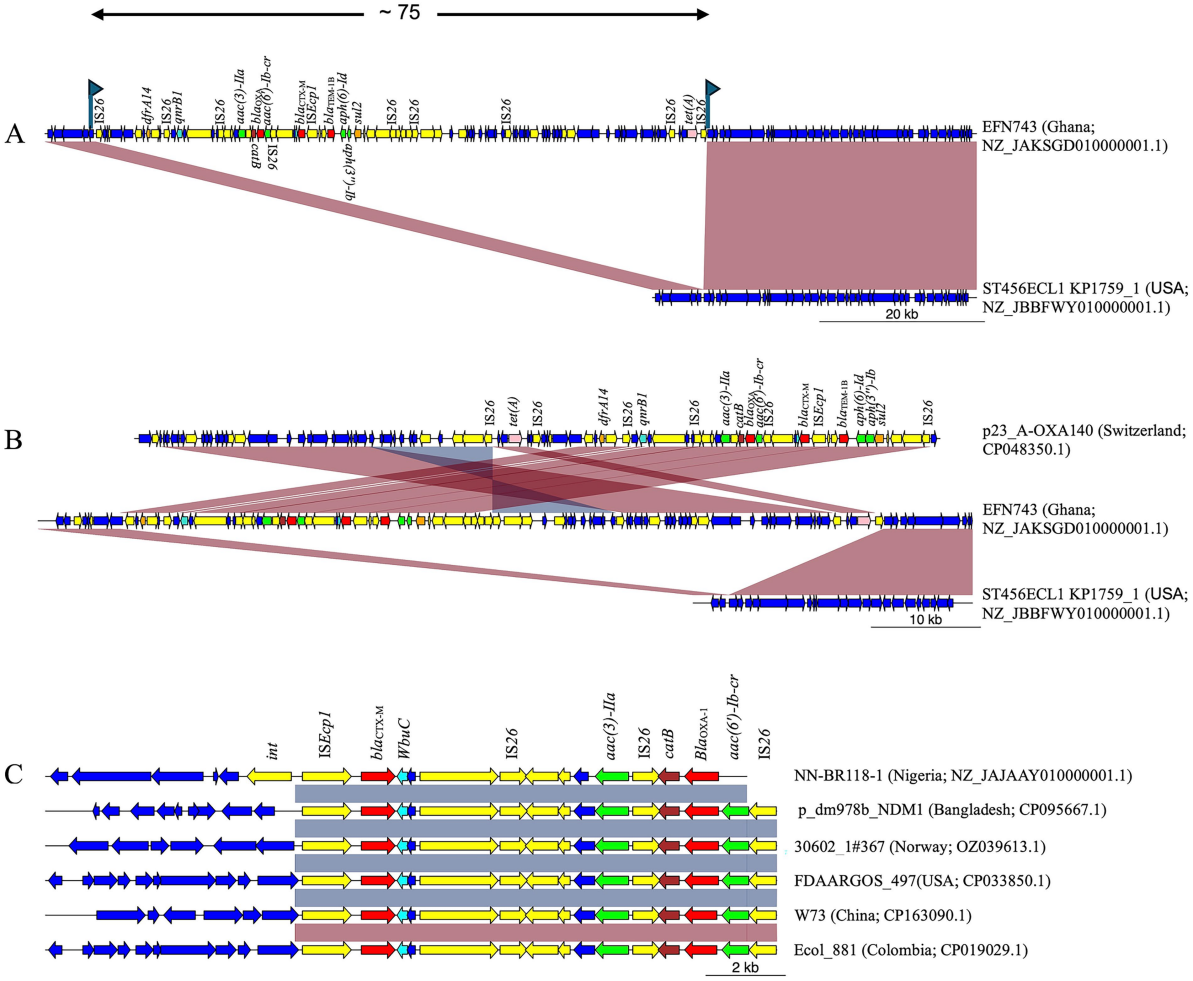
MDR chromosomal insertions in *E. cloacae* strains from West Africa. **(A)** Strain EFN743 from Ghana harbored *bla*_CTX-M_ on a ~ 75 kbp IS26-flanked chromosomal insertion containing multiple AMR genes. The insertion was bracketed by putative 8 bp TSDs (represented by flags). **(B)** The insertion in strain EFN743 was similar to plasmid p23_A-OXA140 from Switzerland, though the gene arrangement was different. **(C)** Nigerian strain NN-BR118-1 carried *bla*_CTX-M_ on a ~ 13 kbp MDR insertion bounded by the int. gene on one end. This insertion was found in several sequences, though they lacked the *int* gene upstream, but harbored IS26 on the opposite end. Yellow; mobile genetic elements. Red; β-lactamase gene. Cyan; *wbuC*. Green; aminoglycoside resistance gene. Brown; chloramphenicol resistance gene. Pink; tetracycline resistance gene. Orange; folate pathway antagonist.

Of the four *E. cloacae* strains, two STs (ST456 and ST544) were identified, while the remaining two could not be typed due to the absence of the seven housekeeping genes (Supplementary Table S4). All four strains carried IS*Ecp1* upstream of *bla*_CTX-M_ and *wbuC* downstream. However, two strains (50%) carried additional AMR genes near *bla*_CTX-M_. For instance, strain EFN743 (ST456, Ghana) possessed genes conferring resistance to trimethoprim (*dfrA14*), quinolones [*qnrB1, aac(6′)-Ib-cr*], aminoglycosides [*aac(3)-IIa*, *aac(6′)-Ib-cr*, *aph(6)-Id*, *aph(3″)-Ib*, *ant(3″)-Ia*], chloramphenicol (*catB*), *β*-lactams (*bla*_OXA-1_, *bla*_TEM-1B_), sulfonamides (*sul2*), and tetracycline (*tetA*). When compared to *E. cloacae* ST456 (ST456ECL1 KP1759_1, Accession Number NZ_JBBFWY010000001.1), these genes were located within a 75,439 bp chromosomal insertion flanked by directly oriented IS26 (Figure 3A). The two peripheral IS26 copies were flanked by putative 8 bp TSDs (GACCACAC) at positions 66,296–66,301 and 141,741–141,748. Notably, rearranged versions were observed in GeneBank sequences, including plasmid p23_A-OXA140 (Accession Number CP048350.1) (Figure 3B). Furthermore, the abovementioned insertion harbored multiple genomic islands rich in virulence factors, mobile elements, and AMR genes (Supplementary Figure S3).

Meanwhile, a comparison of strain NN-BR118-1 (ST544, Nigeria) to *E. cloacae* ATCC13047 (Accession Number CP001918.1) showed that NN-BR118-1 had the *bla*_CTX-M_ gene on an insertion >13 kbp at position 4,685,679–4,698,752 bp. This insertion contained a site-specific integrase gene positioned immediately upstream of IS*Ecp1*, and an 8,284 bp genomic island downstream of the *bla*_CTX-M_ gene. This genomic island included *bla*_OXA_, as well as genes for aminoglycoside [*aac(3)-IIa* and *aac(6′)-Ib-cr*], quinolone [*aac(6′)-Ib-cr*], and chloramphenicol (*catB*) resistance (Supplementary Figure S4). A BLAST analysis of the 13 kbp insertion revealed >100 high-identity matches (>99.9% identity, >11.5 kbp sequence alignment), although none retained the site-specific integrase gene, suggesting rearrangement via IS*Ecp1* or IS26 located on the opposite end (Figure 3C).

### Clonally related *K. pneumoniae* strains share STs and AMR genes

**Figure 4:**
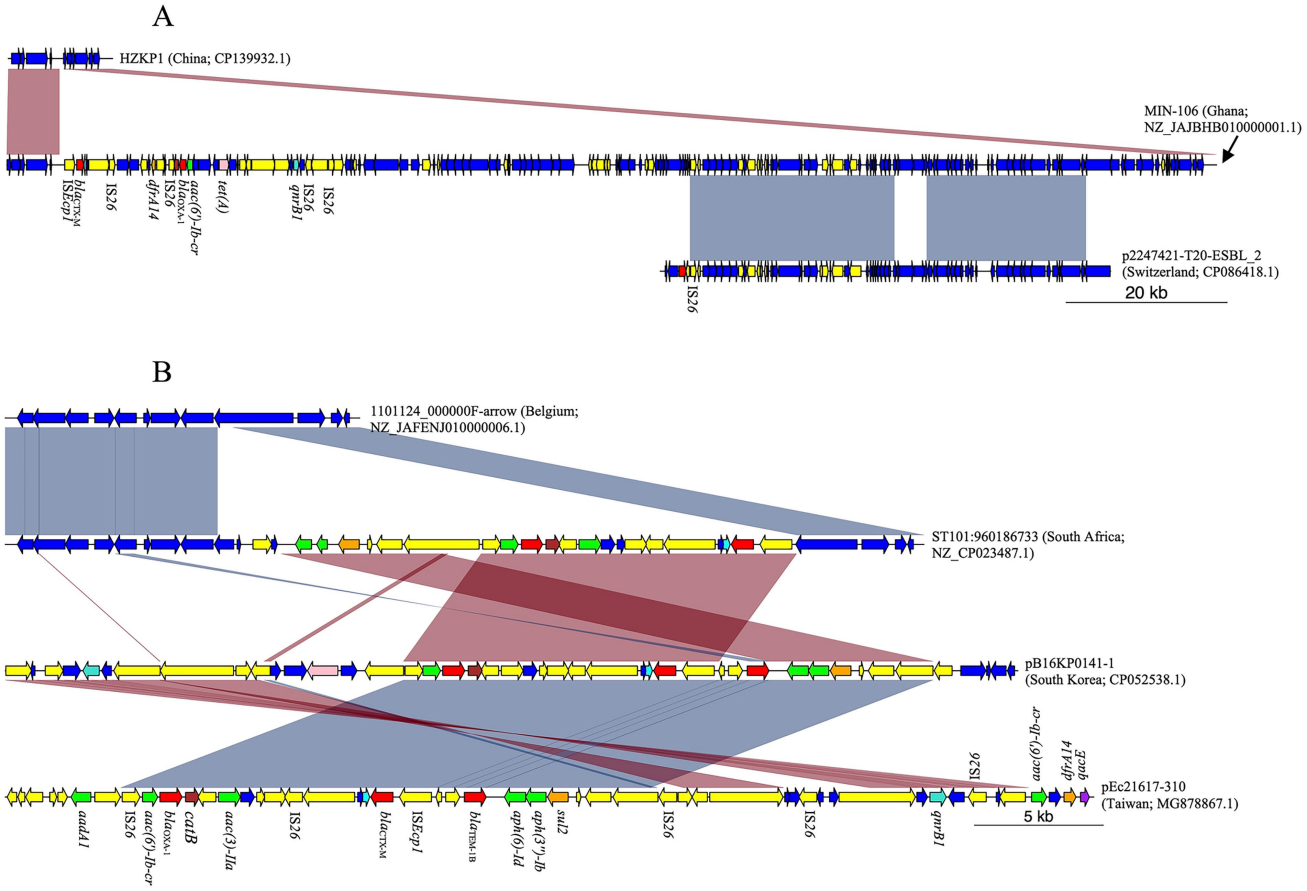
MDR chromosomal insertions in *K. pneumoniae* strains from West Africa. **(A)** Ghanaian strain MIN-106 possessed a 139,735 bp chromosomal insertion bounded by IS*Ecp1*/*bla*_CTX-M-15_/*wbuC* on one end. About 60,300 bp of this insertion was similar to plasmid p2247421-T20-ESBL_2, but the aligned regions did not include any AMR genes. **(B)** South African strain ST101:960186733 harbored multiple AMR genes on a 22,270 bp insertion bounded by IS*Ecp1*/*bla*_CTX-M-15_/*wbuC* on one end. The insertion was similar to the AMR gene cluster, which was observed in plasmids pB16KP0141–1 and pEc21617-310.

Chromosomally integrated *bla*_CTX-M_ was identified in *K. pneumoniae* strains from Ghana, South Africa, Uganda, and Tanzania (Table 1). Ghana strains EFN299 (Accession Number NZ_CP092589.1) and MIN-106 (Accession Number NZ_JAJBHB010000001.1) belonged to ST11 and ST152, respectively (Supplementary Table S5). In addition to the IS*Ecp1*/*bla*_CTX-M-15_/*wbuC* unit, the chromosome of strain EFN299 possessed *oqxA*, *oqxB*, *bla*_SHV-182_, and *fosA6*. However, none of these genes were in the immediate vicinity (i.e., within 10,000 bp) of *bla*_CTX-M_. In contrast, MIN-106 possessed *dfrA14* 8,616 bp downstream of *bla*_CTX-M-15_, along with mobile elements and additional AMR genes [*bla*_OXA-1_, *aac(6′)-Ib-cr*, and *tet(A)*]. When compared to another *K. pneumoniae* ST152 (strain HZKP1, Accession Number CP139932.1), MIN-106 showed a 139,735 bp chromosomal insertion bounded by IS*Ecp1*/*bla*_CTX-M-15_/*wbuC* on one end. BLAST analysis of this insertion, filtered for matches with “percent identity > 99%” and “alignment length > 60,000,” yielded 24 hits. However, these alignments covered approximately 60,300 bp of the insertion. For instance, p2247421-T20-ESBL_2, an IncF plasmid from a clinical ESBL-producing *K. pneumoniae* isolate in Switzerland, showed 60,380 bp of aligned sequence. Notably, these aligned regions did not include the AMR genes observed in MIN-106 (Figure 4A). However, the entire insertion in MIN-106 included intermittently distributed genomic islands associated with virulence, mobility, and metal resistance (Supplementary Figure S5).

From South Africa, *K. pneumoniae* strains were assigned to ST101 (*n* = 1) and ST2497 (*n* = 3), all of which carried *bla*_CTX-M-15_ flanked by upstream IS*Ecp1* and downstream *wbuC*. The three ST2497 strains, KLEB-CRE-TBH-0080 (Accession Number NZ_JAWQUU010000001.1), KLEB-CRE-M09-0012 (Accession Number NZ_JAWQWY010000001.1), and KLEB-CRE-M09-0005 (Accession Number NZ_JAWQXC010000001.1) harbored an identical AMR gene repertoire (*oqxA*, *oqxB*, *bla*_SHV-182_, *fosA*), but none of these genes was in the immediate vicinity of *bla*_CTX-M-15_. In contrast, ST101:960186733 strain (ST101, Accession Number NZ_CP023487.1) possessed the aminoglycoside resistance gene *aac(3)-IIa* positioned 5,153 bp downstream of the *bla*_CTX-M-15_ gene, followed by other AMR genes including *catB*, *bla*_OXA-1_, *aac(6′)-Ib-cr*, *sul2*, *aph(3″)-Ib*, and *aph(6)-Id*. Comparative analysis with another *K. pneumoniae* ST101 genome (1101124_000000F-arrow, Accession Number NZ_JAFENJ010000006.1) revealed that ST101:960186733 harbored the *bla*_CTX-M_ gene on a 22,270 bp insertion bounded by IS*Ecp1* on one end (Figure 4B) and a 20,394 bp genomic island immediately downstream of IS*Ecp1* (Supplementary Figure S6). BLAST analysis of this insertion revealed several plasmids with >85% coverage and 100% sequence identity. Notably, the AMR gene cluster was also identified in plasmids pB16KP0141–1 (IncF, Accession Number CP052538.1) and pEc21617-310 (IncH, Accession Number MG878867.1) (Figure 4B).

Four *K. pneumoniae* strains from Uganda were assigned to ST39 (*n* = 2), ST231 (*n* = 1), and ST1119 (*n* = 1) (Supplementary Table S5). All carried the IS*Ecp1*/*bla*_CTX-M-15_/*wbuC* unit. Two strains, both annotated as MAKM-3381 but with distinct accession numbers and genome lengths, were designated as MAKM-3381A (ST39, Accession Number CP129122.1) and MAKM-3381B (ST1119, Accession Number CP129541.1) to distinguish them. Along with MAKM-5490 (ST39, Accession Number CP130492.1), these strains shared an identical resistance architecture featuring *bla*_TEM-1B_ located 2,821 bp upstream of *bla*_CTX-M-15_ in the opposite orientation. Immediately downstream of *bla*_TEM-1B_ was the aminoglycoside-encoding gene *aac(3)-IIa*. These AMR genes existed on a 45,632 bp genomic island containing virulence genes encoding the type VI secretion system (*hcp*, *tssL*, and *tssK*), mercury resistance (*merR*, *merT*, *merP*, *merC*, *merA*), and various IS elements (Supplementary Figure S7). Notably, this genomic island also exhibited the *repA* gene that encodes the IncFII family plasmid replication initiator RepA.

On the other hand, MAKM-RS081 (ST231, Accession Number CP129536.1) lacked *bla*_TEM-1B_ and did not possess any additional AMR genes in the vicinity of *bla*_CTX-M-15_. Both *K. pneumoniae* strains from Tanzania belonged to ST437 and carried *bla*_CTX-M-15_ flanked by upstream IS*Ecp1* and downstream *wbuC*. No additional AMR genes were identified in the vicinity of the ESBL gene.

Among the 17 *K. pneumoniae* strains from Sudan, two (S-P-N-044.01, Accession Number CP092693.1 and S-P-C-024.01, Accession Number CP092807.1) were assigned to ST11, while the remaining 15 could not be definitively typed due to partial or low-identity alleles in the MSLT housekeeping genes. Yet, all strains carried IS*Ecp1* upstream of *bla*_CTX-M_ but lacked the downstream *wbuC* element. Instead, all the strains featured the IS903B insertion sequence immediately downstream of *bla*_CTX-M-14_. In addition, all the strains harbored several resistance genes, including *bla*_SHV-182_*, bla*_TEM-1B_, and the carbapenemase-encoding *bla*_KPC-2_, although none of these were co-localized with *bla*_CTX-M_.

### *bla*_CTX-M_-harboring MDR insertions existed in clonally unrelated strains

**Figure 5:**
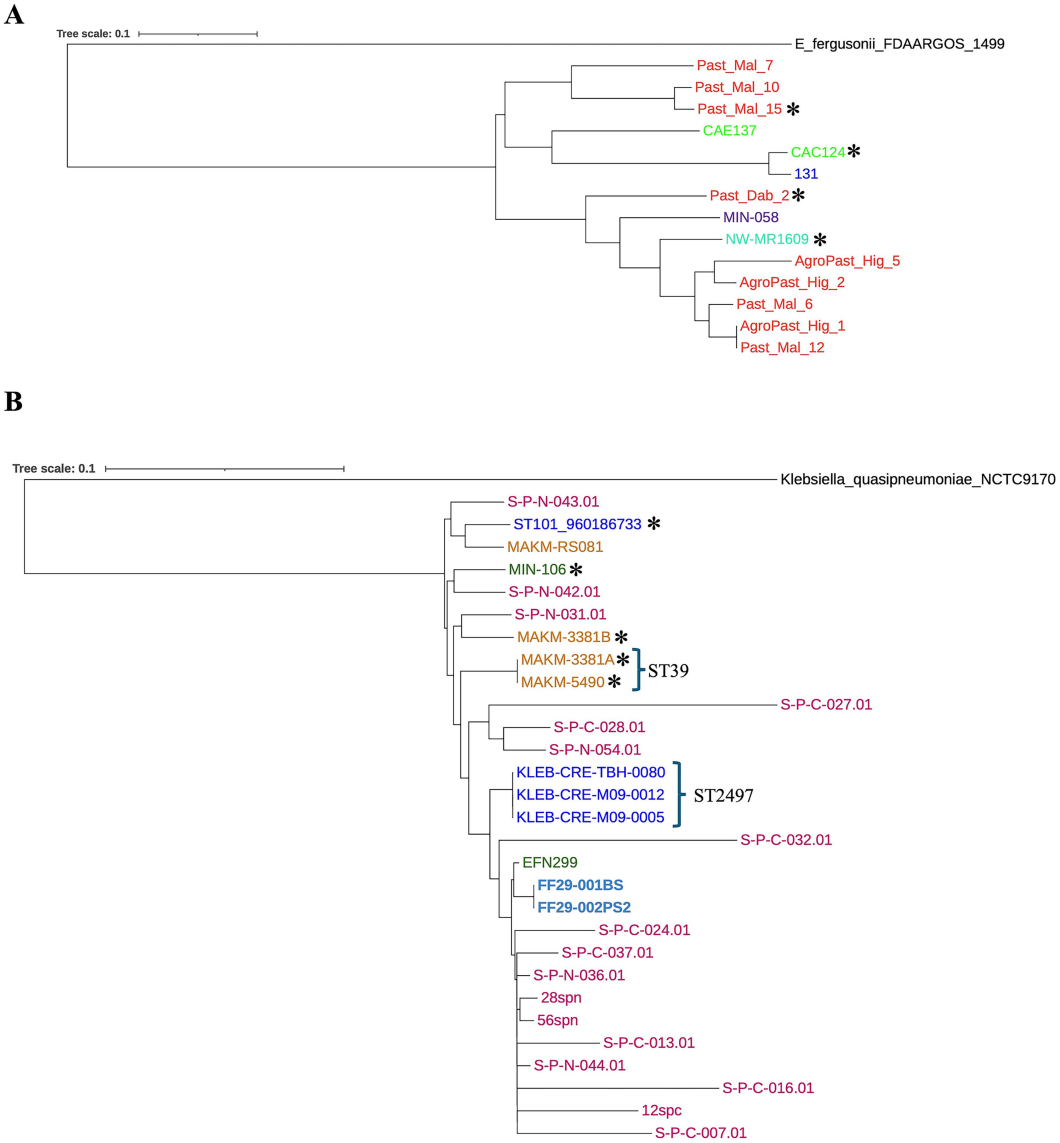
Core-SNP-based phylogenetic trees for *E. coli* **(A)** and *K. pneumoniae* **(B)**. Clustering was seen among strains from the same geographic location. There was no obvious clustering based on the presence large *bla*_CTX-M_-carrying MDR chromosomal insertions. Strains from the same country are indicated by the same color. Asterisks (*) represent the presence of large *bla*_CTX-M_-carrying MDR chromosomal insertions.

Analysis of Core-SNP-based phylogenetic trees showed a tendency for regional clustering, while the presence of *bla*_CTX-M_-harboring MDR insertions was not related to strain clonality (Figure 5). For instance, *E. coli* strains from Ethiopia formed two distinct clades, and Malawi strains were closely related (Figure 5A). Yet, *E. coli* Past_Mal_15 (ST38, Ethiopia) harbored the *qnrS1* gene on a large *bla*_CTX-M_-carrying insertion, while the insertion in the clonally related *E. coli* Past_Mal_10 (ST38, Ethiopia) (Figure 5A) lacked AMR genes other than *bla*_CTX-M_ (Figure 1). Similarly, Malawian *E. coli* strain CAC124 (ST5640) had a 12,837 bp *bla*_CTX-M_-carrying MDR insertion (Figure 1), but its closest relative from South Africa (strain 131) lacked AMR genes around *bla*_CTX-M_. Also, most Sudanese *K. pneumoniae* strains clustered together, while three out of four South African strains formed a clade (Figure 5B). South African *K. pneumoniae* strain ST101:960186733, with a 22,270 bp *bla*_CTX-M_-harboring MDR insertion, was closely related to Ugandan strain MAKM-RS081 (ST231) (Figure 5B), which had no AMR genes near *bla*_CTX-M_. These discrepancies were also observed for closely related *K. pneumoniae* strains MAKM-3381B (with a large *bla*_CTX-M_-carrying insertion) and S-P-N-031.01 (lacking a large *bla*_CTX-M_-carrying insertion), as well as MIN-106 (with a large *bla*_CTX-M_-carrying insertion) and S-P-N-042.01 (lacking a large *bla*_CTX-M_-carrying insertion).

## Discussion

In this study, we utilized publicly available WGS data to investigate the burden and genetic context of chromosomally integrated *bla*_CTX-M_ genes among *Enterobacteriaceae* species from Africa. Chromosomal *bla*_CTX-M_ was detected in ~16% of genomes analyzed, spanning nine out of 18 countries. The most frequently identified allele was *bla*_CTX-M-15_, found in two-thirds of the sequences. The *bla*_CTX-M_ gene was located within the chromosomal insertions flanked by IS*Ecp1* or IS26 elements, highlighting the central role of these mobile elements in facilitating stable chromosomal integration of this clinically significant ESBL determinant. Notably, some of these insertions also harbored additional AMR genes, underscoring their potential to encode chromosomal MDR.

The high prevalence of chromosomal *bla*_CTX-M_ may reflect strong selection pressure arising from widespread antimicrobial use in clinical settings. Chromosomal integration offers a potential evolutionary advantage by reducing fitness costs and structural instability typically associated with plasmid carriage, as well as mitigating the risk of plasmid loss through segregational events during cell division (Gu et al., 2015). The strains examined in this study appear to have acquired secondary chromosomal integration of plasmid-derived *bla*_CTX-M_ via two main mechanisms. In the majority of sequences, the IS*Ecp1* gene was located upstream of *bla*_CTX-M_, consistent with IS*Ecp1*-mediated transposition (Poirel et al., 2005). The presence of MDR islands bounded by IS*Ecp1* further suggests that this element can transpose large DNA segments. These MDR islands often included additional IS elements, indicating that chromosomal integration may occur through a multistep process involving successive transposition events. Nevertheless, the identification of nearly identical insertions in both plasmid and chromosomal sequences supports the possibility of a single-step integration.

Notably, in strains lacking IS*Ecp1*, insertions were flanked by IS26 elements. Previous studies have shown that IS26-mediated transposition can occur via a cointegration mechanism, leading to duplication of IS26 and the formation of 8 bp TSDs (Harmer et al., 2014). Alternatively, a single IS26 element with adjacent “passenger” genes can form a translocatable unit, which may insert next to another IS26 copy through conservative transposition that does not generate TSDs (Harmer et al., 2014). We identified 8 bp TSDs flanking two directly oriented IS26 elements on either end of a 75 kbp chromosomal insertion encoding MDR. This observation is consistent with IS26-mediated replicative transposition. However, comparison with matching GeneBank sequences revealed notable differences in the arrangement of genes within these insertions. Given the presence of multiple IS copies across the segment, it is likely that the genes were integrated through a multistep process involving several mobile gene elements. We speculate that the initial event may have been a copy-in transposition involving a single IS26 element and associated genes, leading to the formation of a second IS26 and TSDs. This could have been followed by a series of conservative (non-replicative) transposition events, whereby translocatable units targeted pre-existing IS26 sites within the growing composite island (Harmer and Hall, 2024).

Meanwhile, in one *E. cloacae* strain, the MDR insertion was flanked by *int* immediately upstream of IS*Ecp1*, suggesting that chromosomal integration may have occurred via site-specific recombination. However, the involvement of IS*Ecp1* cannot be excluded, given its role in mobilizing resistance elements. Notably, this MDR segment was detected in numerous plasmid and chromosome sequences in the GeneBank, all of which lacked the flanking *int* gene but possessed IS*Ecp1* and IS26 on opposite ends. This pattern suggests that structural rearrangement, potentially mediated by IS*Ecp1* and/or IS26, may have occurred following initial integration.

The observation of similar genetic architectures shared by clonally unrelated strains highlights the frequency with which chromosomal integration occurs by horizontal gene transfer. For instance, the *qnrS1* gene was found precisely 4,640 bp downstream of *bla*_CTX-M_ in two genetically distinct *E. coli* strains (ST38 and ST8130), as well as a strain previously identified as *S. sonei* but reassigned to *E. coli* ST484. This suggests that an identical DNA segment was horizontally acquired by diverse genetic backgrounds. In parallel, we also observed evidence of the spread of chromosomal *bla*_CTX-M_ by clonal expansion. Three of the four *K. pneumoniae* strains from South Africa belonged to the same ST (2497) and carried the same AMR profiles. These three strains were recovered from patients admitted to the same hospital in Cape Town (Marais et al., 2024), supporting a likely scenario of patient-to-patient transmission. Similarly, the two *K. pneumoniae* ST39 from Uganda and the two ST437 strains from Tanzania shared indistinguishable resistance gene arrangements, consistent with local clonal dissemination. In Sudan, while only two of the 17 *K. pneumoniae* strains were typed (both as ST11), the remaining 15 untyped strains also harbored the same set of AMR genes, suggesting either the expansion of a single dominant clone or recurrent horizontal acquisition of a common resistance island by unrelated strains. Generally, most strains from different countries did not share STs, and when the same ST was observed across borders, the associated AMR profiles differed. For example, *K. pneumoniae* ST11 from Ghana and Sudan had *bla*_CTX-M-15_ and *bla*_CTX-M-14_, respectively, and the two strains exhibited distinct resistance architectures, indicating independent acquisition events and no evidence of inter-country clonal spread. Phylogenetic analysis revealed regional clustering of *E. coli* and *K. pneumoniae* strains, but the presence of *bla*_CTX-M_-harboring MDR insertions did not correlate with strain clonality, highlighting the role of horizontal gene transfer in MDR dissemination.

Interestingly, the MDR region identified in the Malawian strain CAC124 (ST5640) was detected in the chromosomes of over 30 *E. coli* ST131 strains (Supplementary Table S3). While CAC124 most likely acquired this segment through horizontal gene transfer, its consistent presence within the ST131 lineage suggests subsequent clonal dissemination. The broad geographic distribution of *E. coli* ST131 carrying a chromosomally integrated MDR-encoding genetic island could suggest an emerging clonal wave of extraintestinal pathogenic *E. coli*, particularly given that ST131 is already recognized as a pandemic clone.

The presence of multiple genomic islands in the analyzed genomes highlights the high frequency of horizontal gene transfer events and the potential role of factors beyond AMR genes for virulence and fitness enhancement. Furthermore, detecting plasmid replicons on chromosomes confirms the horizontal origins of these factors. While exceptionally large plasmids (>1.7 Mbp) have been reported in rare cases (Kothari et al., 2019), all the replicon-harboring contigs in this study were larger than 4.9 Mbp (Table 2), ruling out their possibility of being plasmids.

This study is not without limitations. First, some strains could not be assigned STs due to missing loci or nucleotide ambiguities within the MLST regions. Second, we assumed that all genomic sequences exceeding 1,000,000 bp represented chromosomal sequences, which may not always be true, as described above. Third, our approach may have excluded chromosomal segments <1,000,000 bp, as many genome assemblies are incomplete. Lastly, a strain previously reported as *S. sonei* by Microflex LT MALDI-TOF MS (Bruker Daltonik, GmbH, United Kingdom) (Portal et al., 2024) was reassigned to *E. coli* ST484 based on our WGS analysis. This discrepancy is not unexpected, as *Shigella* species are phylogenetically nested within *E. coli*, prompting ongoing discussions to reclassify *Shigella* as a sublineage of *E. coli* (Lan and Reeves, 2002).

## Conclusion

We used publicly available WGS data to characterize the genetic landscape of chromosomally integrated *bla*_CTX-M_ among *Enterobacteriaceae* strains from Africa. Approximately 16% of the analyzed genomes carried chromosomally encoded *bla*_CTX-M_, many of which could be linked to plasmid-derived origins. In several cases, the *bla*_CTX-M_-containing chromosomal insertions also harbored additional AMR genes, resulting in genotypically MDR chromosomal segments. While widespread clonal dissemination was observed in the globally dominant *E. coli* ST131 lineage, clonal spread among African strains appeared localized, often limited to individual hospital settings. Our findings underscore an evolutionary strategy by which *Enterobacteriaceae* stabilize and preserve MDR traits through chromosomal integration, potentially ensuring long-term persistence even in the absence of plasmid-mediated transmission.

## Data Availability

The original contributions presented in the study are included in the article/Supplementary material, further inquiries can be directed to the corresponding author.

## Footnotes

^1^ https://github.com/tseemann/mlst

^2^ https://pubmlst.org/
