# Supplementary material for: In silico characterization of chromosomally integrated blaCTX-M genes among clinical Enterobacteriaceae in Africa: insights from whole-genome analysis
Source: Front Microbiol. 2025 Sep 12;16:1655907. doi: 10.3389/fmicb.2025.1655907 (PMC12463934; doi:10.3389/fmicb.2025.1655907)
Supplement: Supplementary file 3 [file Data_Sheet_3.PDF]

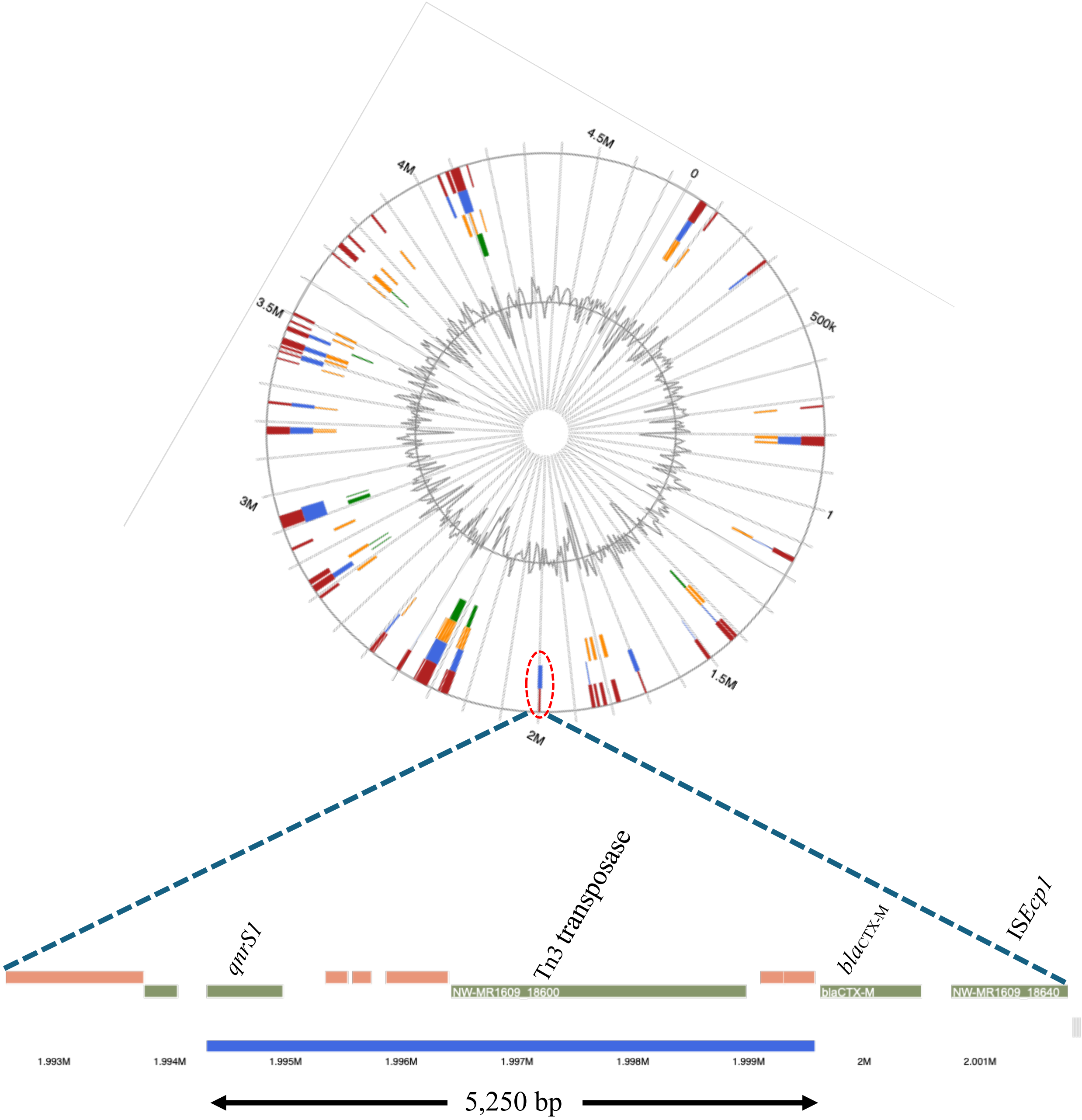

Figure S1. Strain NW-MR1609 (*E. coli* ST484, Nigeria). The circular map shows multiple genomic islands across the genome. A zoomed section of the region flanking the $bla_{\text{CTX-M}}$ gene shows an adjacent 5250 bp genomic island carrying the *qnrS1* and Tn3-like element Tn3 family transposase genes. Colors in the circular map represent the prediction methods for genomic islands:

Maroon; Integrated. Blue; IslandPath-DIMOB. Orange; SIGI-HMM. Green; IslandPick
