# Supplementary material for: In silico characterization of chromosomally integrated blaCTX-M genes among clinical Enterobacteriaceae in Africa: insights from whole-genome analysis
Source: Front Microbiol. 2025 Sep 12;16:1655907. doi: 10.3389/fmicb.2025.1655907 (PMC12463934; doi:10.3389/fmicb.2025.1655907)
Supplement: Supplementary file 5 [file Data_Sheet_5.PDF]

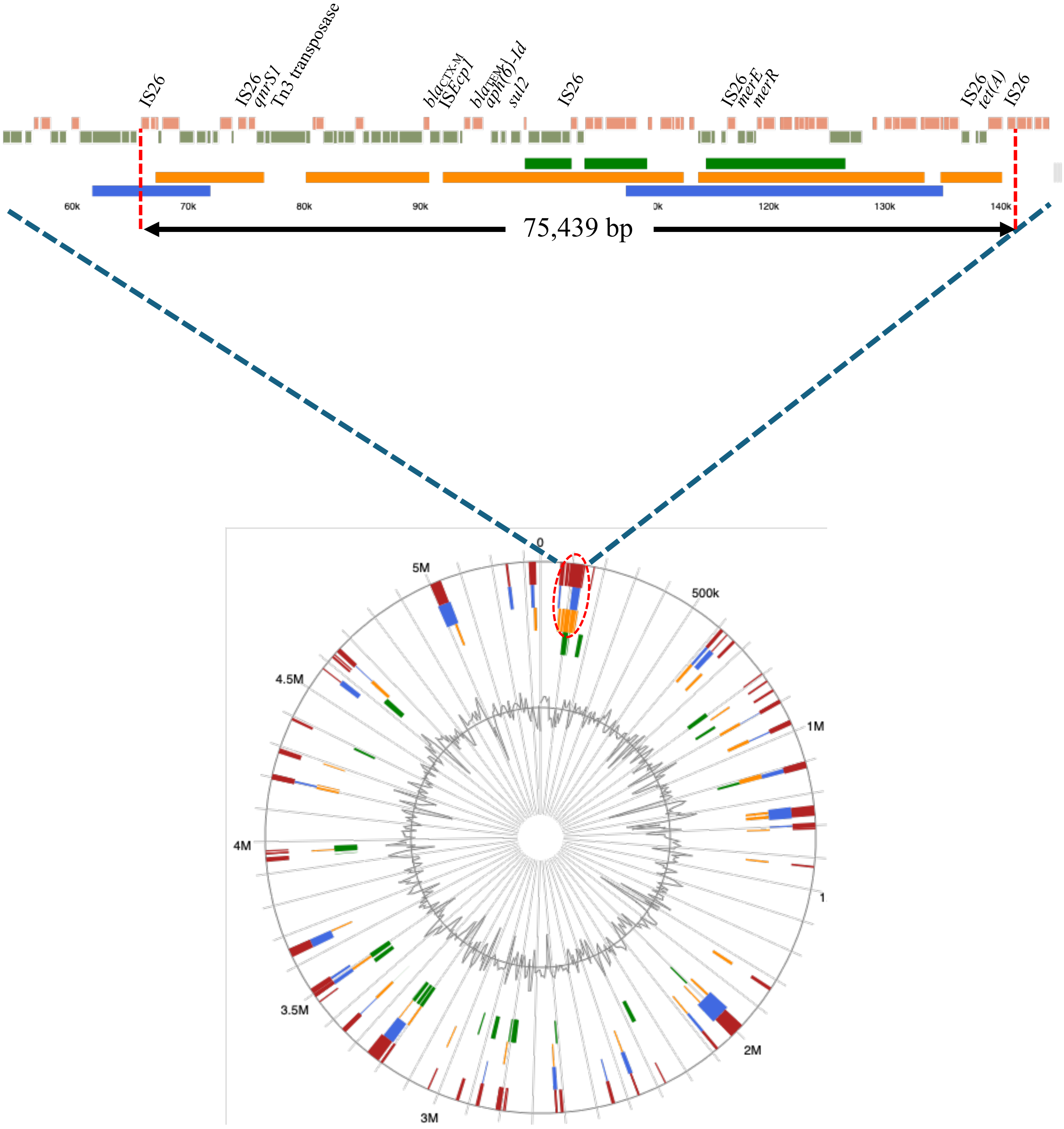

Figure S3. Strain EFN743 (*E. cloacae* ST456, Ghana). The circular map shows multiple genomic islands across the genome. The 75 kbp chromosomal insertion contained multiple genomic islands carrying AMR genes and transposable elements.

Colors in the circular map represent the prediction methods for genomic islands:

Maroon; Integrated. Blue; IslandPath-DIMOB. Orange; SIGI-HMM. Green; IslandPick
