# Supplementary material for: In silico characterization of chromosomally integrated blaCTX-M genes among clinical Enterobacteriaceae in Africa: insights from whole-genome analysis
Source: Front Microbiol. 2025 Sep 12;16:1655907. doi: 10.3389/fmicb.2025.1655907 (PMC12463934; doi:10.3389/fmicb.2025.1655907)
Supplement: Supplementary file 6 [file Data_Sheet_6.PDF]

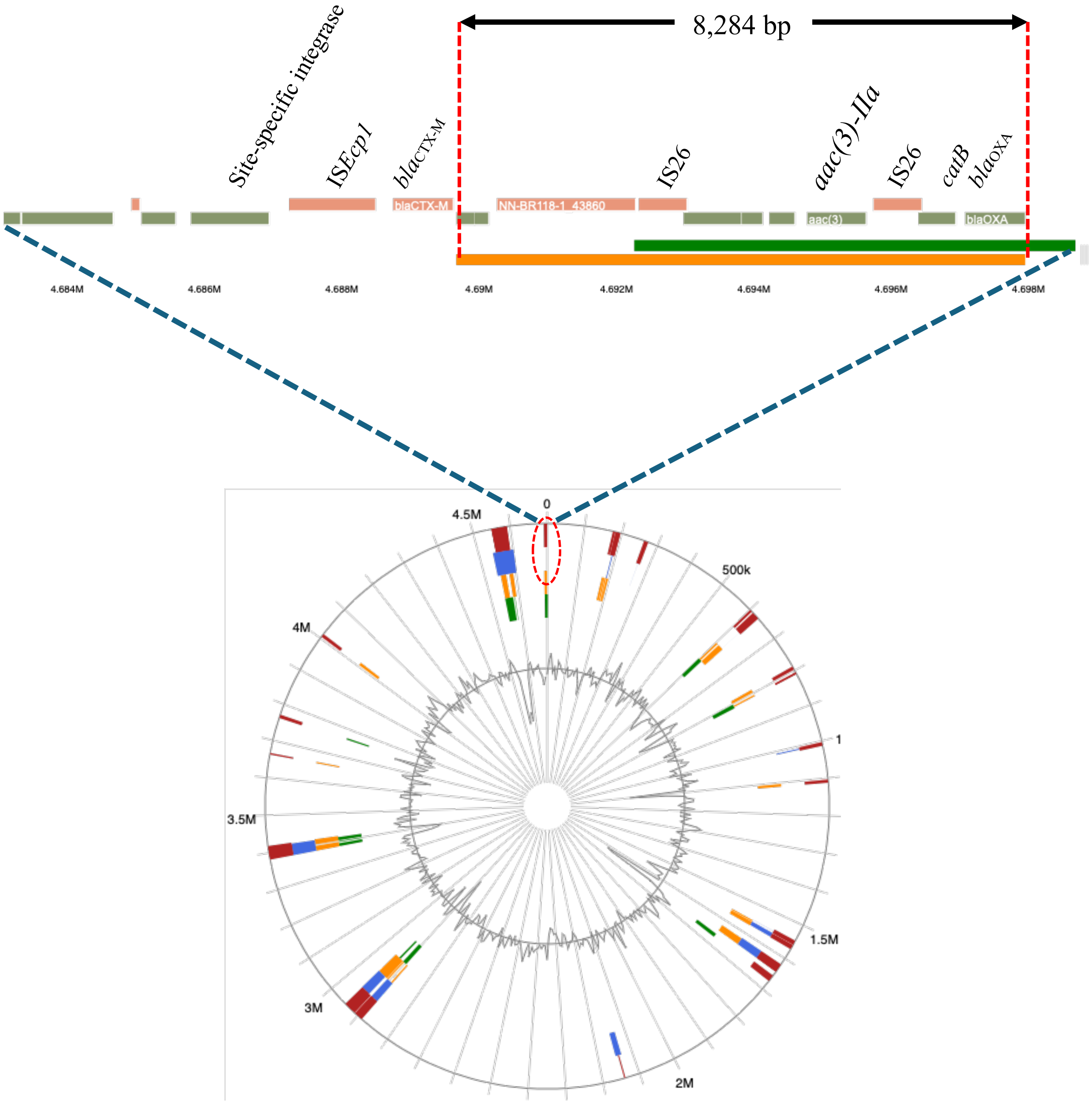


Figure S4. Strain NN-BR118-1 (*E. cloacae* ST544, Nigeria). The *bla*$_{\text{CTX-M}}$-carrying insertion included an 8 kbp genomic island harboring IS elements and AMR genes.

Colors in the circular map represent the prediction methods for genomic islands: Maroon; Integrated. Blue; IslandPath-DIMOB. Orange; SIGI-HMM. Green; IslandPick
