# Supplementary material for: In silico characterization of chromosomally integrated blaCTX-M genes among clinical Enterobacteriaceae in Africa: insights from whole-genome analysis
Source: Front Microbiol. 2025 Sep 12;16:1655907. doi: 10.3389/fmicb.2025.1655907 (PMC12463934; doi:10.3389/fmicb.2025.1655907)
Supplement: Supplementary file 7 [file Data_Sheet_7.PDF]

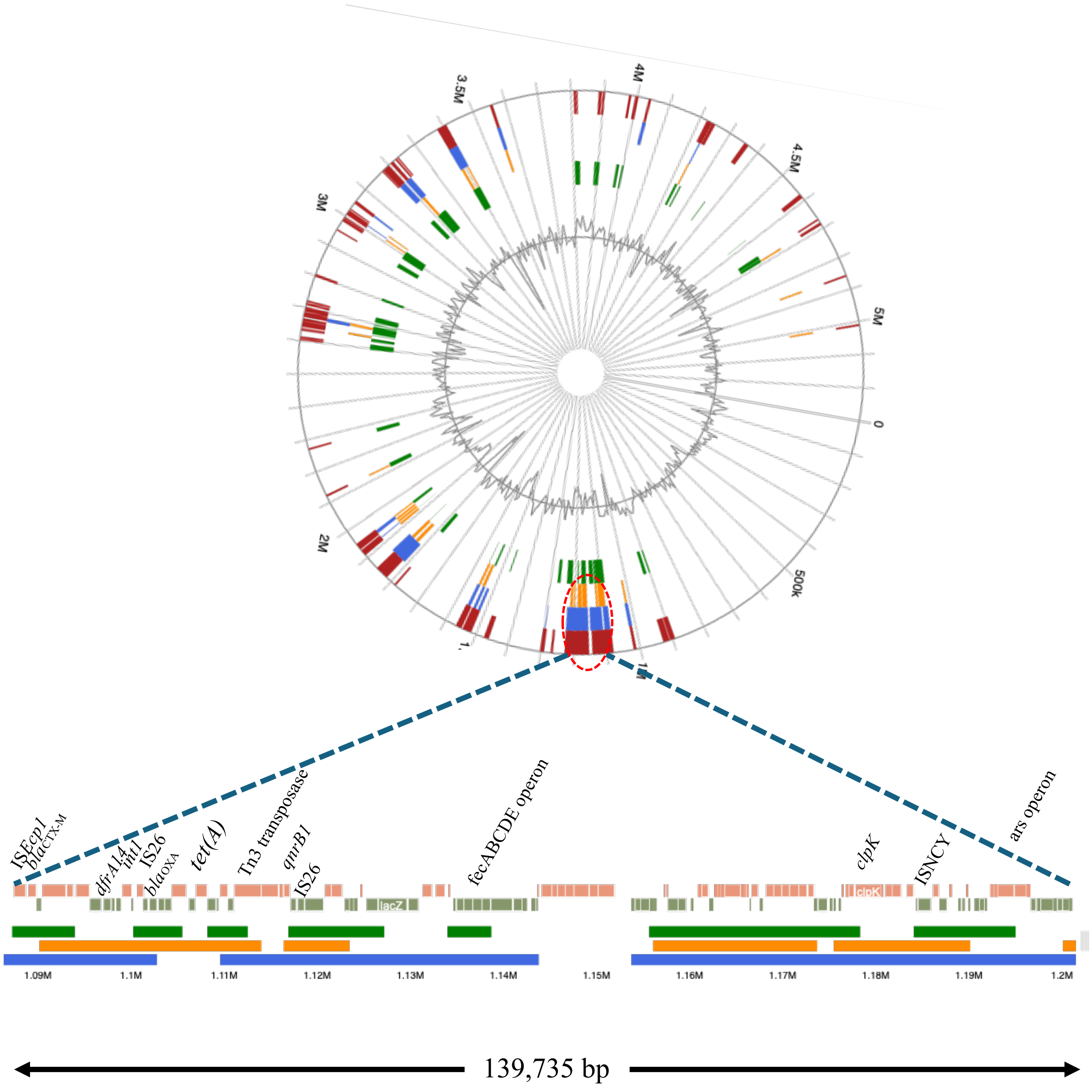

Figure S5. Strain MIN-106 (*K. pneumoniae* ST152, Ghana). The $bla_{CTX\text{-}M}$-carrying insertion included various genomic islands across its entire length.

Colors in the circular map represent the prediction methods for genomic islands: Maroon; Integrated. Blue; IslandPath-DIMOB. Orange; SIGI-HMM. Green; IslandPick

ISNCY: ISNCY family transposase
