# Supplementary material for: In silico characterization of chromosomally integrated blaCTX-M genes among clinical Enterobacteriaceae in Africa: insights from whole-genome analysis
Source: Front Microbiol. 2025 Sep 12;16:1655907. doi: 10.3389/fmicb.2025.1655907 (PMC12463934; doi:10.3389/fmicb.2025.1655907)
Supplement: Supplementary file 8 [file Data_Sheet_8.PDF]

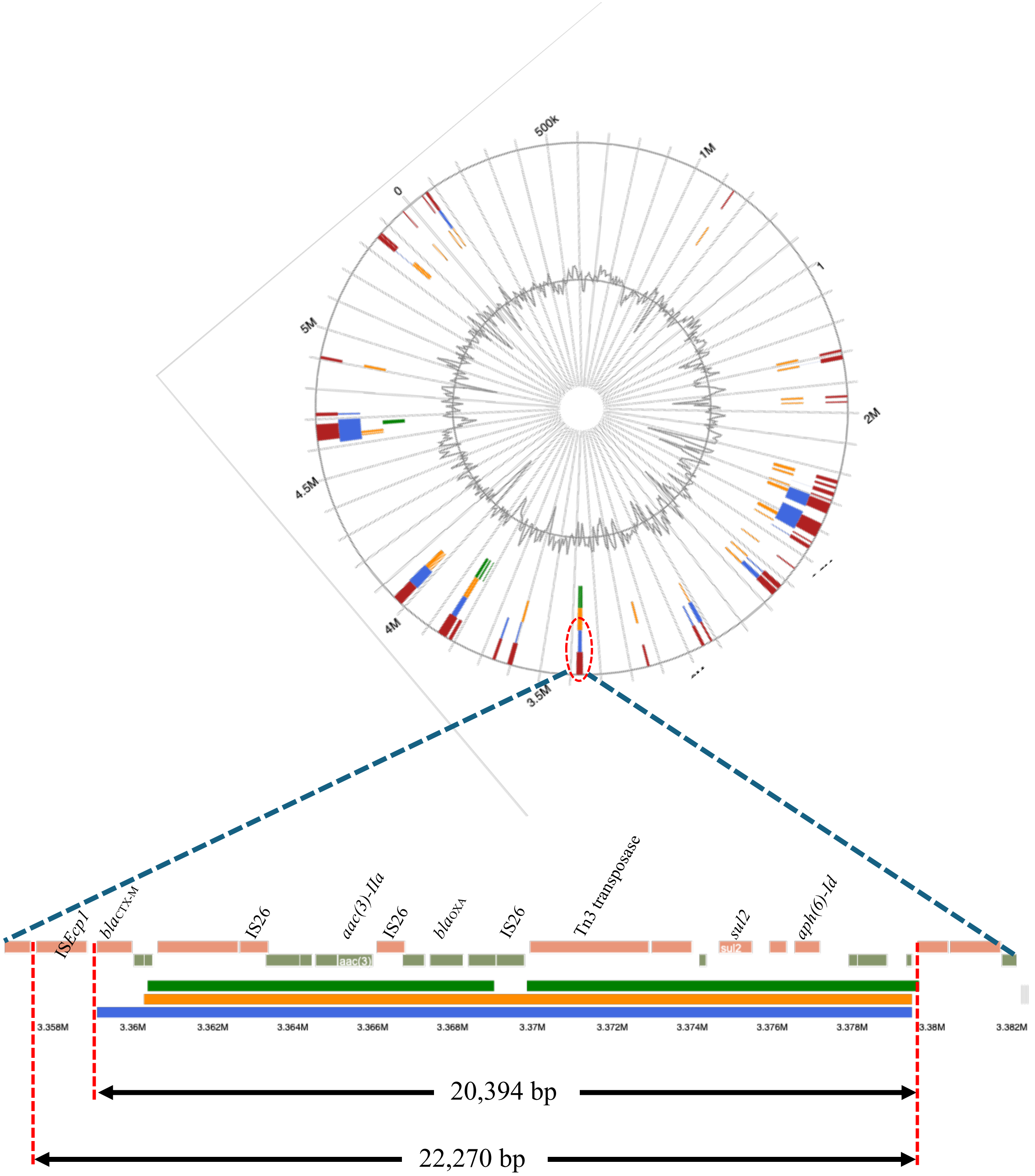

Figure S6. Strain ST101:960186733 (*K. pneumoniae* ST101, South Africa). The *bla*$_{CTX-M}$-carrying insertion included a 20 kbp genomic island harboring IS elements and AMR genes.

Colors in the circular map represent the prediction methods for genomic islands: Maroon; Integrated. Blue; IslandPath-DIMOB. Orange; SIGI-HMM. Green; IslandPick
