# Supplementary material for: In silico characterization of chromosomally integrated blaCTX-M genes among clinical Enterobacteriaceae in Africa: insights from whole-genome analysis
Source: Front Microbiol. 2025 Sep 12;16:1655907. doi: 10.3389/fmicb.2025.1655907 (PMC12463934; doi:10.3389/fmicb.2025.1655907)
Supplement: Supplementary file 9 [file Data_Sheet_9.PDF]

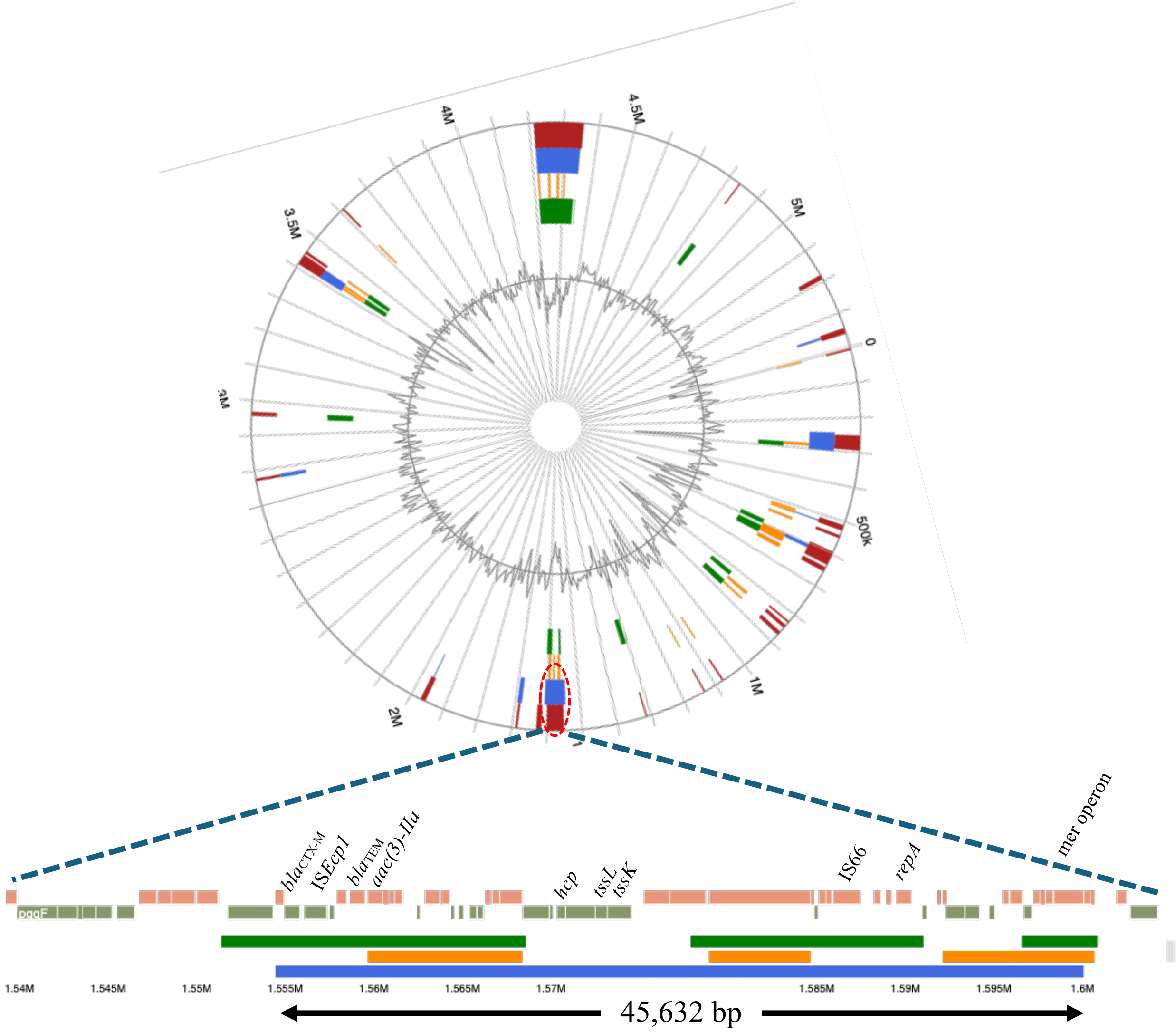

Figure S7. MAKM-5490 (*K. pneumoniae* ST39). These *bla*$_{\text{CTX-M}}$ gene existed on a 45.6 kbp genomic island carrying virulence genes encoding the type VI secretion system and mercury resistance. Colors in the circular map represent the prediction methods for genomic islands:

Maroon; Integrated. Blue; IslandPath-DIMOB. Orange; SIGI-HMM. Green; IslandPick
