# Supplementary material for: In silico characterization of chromosomally integrated blaCTX-M genes among clinical Enterobacteriaceae in Africa: insights from whole-genome analysis
Source: Front Microbiol. 2025 Sep 12;16:1655907. doi: 10.3389/fmicb.2025.1655907 (PMC12463934; doi:10.3389/fmicb.2025.1655907)
Supplement: Supplementary file 10 [file Table_1.DOCX]

Table S2. E. coli and Shigella chromosomes carrying the bla_CTX-M_ gene

| Country | Strain ID | ST | Accession Number | AMR genes |
| --- | --- | --- | --- | --- |
| Malawi | CAC124 | 5640 | NZ_JAWZSZ010000002.1 | *bla*_CTX-M-15_, *mdf(A)*, *aac(6')-Ib-cr*, *bla*_OXA-1_, *aac(3)-IIa* |
|  | CAE137 | - | NZ_JAWZTC010000001.1 | *bla*_CTX-M-55_, *mdf(A)* |
| Niger | MIN-058 | 11025 | NZ_JAJBIA010000001.1 | *bla*_CTX-M-55_, *mdf(A)* |
| Nigeria | NW-MR1609^s^ | 484 | NZ_JASATV010000001.1 | *bla*_CTX-M-15_, *qnrS1*, *mdf(A)* |
| South Africa | 131 | 131 | NZ_CP093011.1 | *bla*_CTX-M-27_, *mdf(A)*, *dfrA17*, *aadA5*, *sul1*, *mph(A)*, *aph(6)-Id*, *aph(3'')-Ib*, *sul2* |
| Ethiopia | Past_Mal_15 | 38 | NZ_JBFOAP010000001.1 | *bla*_CTX-M-15_, *qnrS1*, *mdf(A)* |
|  | AgroPast_Hig_5 | - | NZ_JBFOAQ010000002.1 | *bla*_CTX-M-15_, *mdf(A)* |
|  | Past_Mal_12 | 450 | NZ_JBFOAT010000002.1 | *bla*_CTX-M-15_, *mdf(A)* |
|  | Past_Mal_10 | 38 | NZ_JBFOAZ010000001.1 | *bla*_CTX-M-14_, *mdf(A)*, *bla*_TEM-1_, *tet(D)*, *aph(3')-Ia*, *aph(6)-Id*,  *aph(3'')-Ib*, *sul2*,  *ant(3'')-Ia*, *dfrA1* |
|  | Past_Mal_7 | 8131 | NZ_JBFOBO010000002.1 | *bla*_CTX-M-15_, *mdf(A)* |
|  | Past_Mal_6 | 48 | NZ_JBFOBQ010000001.1 | *bla*_CTX-M-15_, *tet(B)*,  *bla*_TEM-1_, *aac(3)-IId*,  *dfrA12*, *aadA2*, *sul1*,  *mph(A)*, *catA1*, *mdf(A)* |
|  | Past_Dab_2 | 8130 | NZ_JBFOBS010000001.1 | *bla*_CTX-M-15_, *qnrS1*, *mdf(A)*, *ant(3'')-Ia*, *dfrA1* |
|  | AgroPast_Hig_2 | 227 | NZ_JBFOCD010000002.1 | *bla*_CTX-M-15_, *mdf(A)*, *ant(3'')-Ia*, *dfrA1* |
|  | AgroPast_Hig_1 | 450 | NZ_JBFOCF010000001.1 | *bla*_CTX-M-15_ |

^s^*S. sonei*
