# Supplementary material for: In silico characterization of chromosomally integrated blaCTX-M genes among clinical Enterobacteriaceae in Africa: insights from whole-genome analysis
Source: Front Microbiol. 2025 Sep 12;16:1655907. doi: 10.3389/fmicb.2025.1655907 (PMC12463934; doi:10.3389/fmicb.2025.1655907)
Supplement: Supplementary file 11 [file Table_2.DOCX]

Table S4. Enterobacter chromosomes carrying the bla_CTX-M_ gene

| Country | Strain ID | ST | Accession Number | AMR genes |
| --- | --- | --- | --- | --- |
| Ghana | EFN743 | 456 | NZ_JAKSGD010000001.1 | \| *bla*_CTX-M-15_, *dfrA14*, *qnrB1*, *aac(3)-IIa*, *bla*_OXA-1_, *aac(6')-Ib-cr*, *bla*_TEM-1B_, *aph(6)-Id*, *aph(3'')-Ib*, *sul2*, *tet(A)*, *oqxA*, *oqxB*, *fosA*, *bla*_CMH-3_, *ant(3'')-Ia*, \| \| --- \| |
| Nigeria | NN-BR118-1 | 544 | NZ_JAJAAY010000001.1 | \| *bla*_CTX-M-15_, *oqxA*, *oqxB*, *tet(A)*, *bla*_TEM-1B_, *sul1*, *ant(3'')-Ia*, *dfrA1*, *bla*_ACT-16_, *fosA*, *aac(3)-IIa*, *bla*_OXA-1_, *aac(6')-Ib-cr* \| \| --- \| |
|  | WS_752 | - | NZ_JARWIJ010000004.1 | *bla*_CTX-M-15_ |
| Egypt | 98230 | - | NZ_JBJGKV010000006.1 | *bla*_CTX-M-15_ |
