# Supplementary material for: In silico characterization of chromosomally integrated blaCTX-M genes among clinical Enterobacteriaceae in Africa: insights from whole-genome analysis
Source: Front Microbiol. 2025 Sep 12;16:1655907. doi: 10.3389/fmicb.2025.1655907 (PMC12463934; doi:10.3389/fmicb.2025.1655907)
Supplement: Supplementary file 12 [file Table_3.DOCX]

Table S5. Klebsiella pneumoniae chromosomes carrying the bla_CTX-M_ gene

| Country | Assigned ID | ST | Accession Number | AMR genes |
| --- | --- | --- | --- | --- |
| Ghana | EFN299 | 11 | NZ_CP092589.1 | *bla*_CTX-M-15_, *oqxA*, *oqxB*, *bla*_SHV-182_, fosA6 |
|  | MIN-106 | 152 | NZ_JAJBHB010000001.1 | *bla*_CTX-M-15_, *dfrA14*, *bla*_OXA-1_, *aac(6')-Ib-cr*, *tet(A)*, *qnrB1*, *oqxA*, *oqxB*, *bla*_SHV-187_, *fosA* |
| South Africa | ST101:960186733 | 101 | NZ_CP023487.1 | *bla*_CTX-M-15_, *aac(3)-IIa*, *bla*_OXA-1_, *aac(6')-Ib-cr*, *sul2*, *aph(3'')-Ib*, *aph(6)-Id*, *bla*_SHV-106_, *fosA* |
|  | KLEB-CRE-TBH-0080 | 2497 | NZ_JAWQUU010000001.1 | *oqxA*, *oqxB*, *bla*_SHV-182_, *bla*_CTX-M-15_, *fosA* |
|  | KLEB-CRE-M09-0012 | 2497 | NZ_JAWQWY010000001.1 | *bla*_CTX-M-15_, *oqxA*, *oqxB*, *bla*_SHV-182_, *fosA* |
|  | KLEB-CRE-M09-0005 | 2497 | NZ_JAWQXC010000001.1 | *bla*_CTX-M-15_, *oqxA*, *oqxB*, *bla*_SHV-182_, *fosA* |
| Uganda | MAKM-3381A | 39 | CP129122.1 | *bla*_CTX-M-15_, *bla*_TEM-1B_, *aac(3)-IIa*, *fosA*, *oqxA*, *oqxB* |
|  | MAKM-RS081 | 231 | CP129536.1 | *bla*_CTX-M-15_, *fosA*, *bla*_SHV-106_, *oqxA*, *oqxB* |
|  | MAKM-3381B | 1119 | CP129541.1 | \| *bla*_CTX-M-15_, tet(A), *bla*_TEM-1B_, aac(3)-IIa, *bla*_SHV-172_, f*osA6*, *sul1*, *ant(3'')-Ia*, *dfrA15*, *oqxA*, *oqxB* \| \| --- \| |
|  | MAKM-5490 | 39 | CP130492.1 | \| *bla*_CTX-M-15_, *bla*_SHV-187_, *bla*_TEM-1B_, *aac(3)-IIa*, *fosA*, *oqxA*, *oqxB* \| \| --- \| |
| Tanzania | FF29-001BS | 437 | CP140473.1 | *bla*_CTX-M-15_, *bla*_SHV-182_, *oqxA*, *oqxB*, *fosA6* |
|  | FF29-002PS2 | 437 | NZ_CP139784.1 | *bla*_CTX-M-15_, *bla*_SHV-182_, *oqxA*, *oqxB*, *fosA6* |
| Sudan | 28spn | - | CP092527.1 | *bla*_CTX-M-14_*, bla*_TEM-1B_*, rmtB, tet(G), floR, aadA2, dfrA12, aac(3)-Iid, aph(6)-Id, aph(3'')-Ib, sul2, bla*_CTX-M-189_ |
|  | 56spn | - | CP092528.1 | *bla*_CTX-M-14_*, bla*_TEM-1B_*, rmtB, tet(G), floR, aadA2, dfrA12, aac(3)-Iid, aph(6)-Id, aph(3'')-Ib, sul2, fosA6, mdf(A)* |
|  | S-P-N-044.01 | 11 | CP092693.1 | *bla*_CTX-M-14_*, bla*_SHV-182_*, fosA6, bla*_TEM-1B_*, bla*_KPC-2_*,* |
|  | S-P-N-036.01 | - | CP092695.1 | *bla*_CTX-M-14_*, bla*_TEM-1B_*, rmtB, tet(G), floR, aadA2, dfrA12, aac(3)-Iid, aph(6)-Id, aph(3'')-Ib, sul2,* *bla*_KPC-2_*, blaTEM-1B, fosA6, bla*_SHV-182_ |
|  | S-P-N-042.01 | - | CP092696.1 | *bla*_CTX-M-14_*, bla*_KPC-2_*, bla*_TEM-1B_*, rmtB, tet(G), floR, aadA2, dfrA12, aac(3)-Iid, aph(6)-Id, aph(3'')-Ib, sul2, bla*_CTX-M-14_*, bla*_SHV-187_*, fosA* |
|  | S-P-N-043.01 | - | CP092697.1 | *bla*_CTX-M-15_*, bla*_KPC-2_*, bla*_TEM-1B_*, rmtB, tet(G), floR,* *ant(3'')-Ia, aac(3)-Iid, aph(6)-Id, aph(3'')-Ib, sul2, bla*_SHV-106_*, fosA6* |
|  | S-P-C-013.01 | - | CP092805.1 | *bla*_CTX-M-55_*, bla*_TEM-1B_*, rmtB, tet(G), floR, aadA2, dfrA12, aac(3)-Iid, aph(6)-Id, aph(3'')-Ib, sul2, bla*_CTX-M-184_*, bla*_SHV-11_*, bla*_KPC-2_ |
|  | S-P-C-007.01 | - | CP092806.1 | *bla*_CTX-M-14_*, bla*_TEM-198_*, rmtB, tet(G), floR, aadA2, dfrA12, aac(3)-Iid, aph(6)-Id, aph(3'')-Ib, sul2, bla*_SHV-11_*, fosA6, bla*_OXA-181_*, bla*_KPC-2_ |
|  | S-P-C-024.01 | 11 | CP092807.1 | *bla*_CTX-M-14_*, bla*_TEM-1B_*, rmtB, tet(G), floR, aadA2, dfrA12, aac(3)-Iid, aph(6)-Id, aph(3'')-Ib, sul2, bla*_SHV-182_*, fosA6, bla*_KPC-2_ |
|  | S-P-C-028.01 | - | CP092808.1 | *bla*_CTX-M-15_*, bla*_TEM-1B_*, rmtB, tet(G), floR, aadA16, dfrA12, aac(3)-Iia, aph(6)-Id, aph(3'')-Ib, sul2, bla*_CTX-M-156_*, bla*_SHV-187_*, fosA6, bla*_KPC-2_ |
|  | S-P-N-031.01 | - | CP092809.1 | *bla*_CTX-M-15_*, bla*_KPC-2_*, bla*_TEM-156_*, bla*_TEM-1A_*, rmtB, tet(G), floR, ant(3'')-Ia, aac(3)-Iia, aph(6)-Id, aph(3'')-Ib, sul2, bla*_SHV-11_*,* |
|  | S-P-C-027.01 | - | CP092810.1 | *bla*_CTX-M-14_*, bla*_TEM-1B_*, rmtB, tet(G), floR, aadA2, dfrA12, aac(3)-Iia, bla*_TEM-1C_*, aph(6)-Id, aph(3'')-Ib, sul2, bla*_CTX-M-156_*, bla*_SHV-182_*, fosA, bla*_KPC-2_ |
|  | S-P-C-032.01 | - | CP092811.1 | *bla*_CTX-M-15,_ *bla*_TEM-1B_*, rmtB, tet(G), floR, aadA2, dfrA12, aac(3)-Iid, aph(6)-Id, aph(3'')-Ib, sul2, bla*_SHV-182_*,* *fosA5, bla*_KPC-2_ |
|  | S-P-C-037.01 | - | CP092812.1 | *bla*_CTX-M-14_*, bla*_TEM-1B_*, rmtB, tet(G), floR, aadA2, dfrA12, aac(3)-IId, bla*_TEM-1B_*, aph(6)-Id, aph(3'')-Ib, sul2, bla*_SHV-187_*, fosA, bla*_KPC-2_ |
|  | S-P-C-016.01 | - | CP092813.1 | *bla*_CTX-M-14_*, bla*_TEM-206_*, rmtB, tet(G), floR, aadA2, dfrA12, aac(3)-Iid, aph(6)-Id, aph(3'')-Ib, sul2, bla*_SHV-11_*, fosA6, bla*_TEM-206_*, bla*_KPC-2_ |
|  | S-P-N-054.01 | - | CP092840.1 | *bla*_CTX-M-15_*, bla*_KPC-2_*, bla*_TEM-1B_*, fosA5, bla*_SHV-187_*, rmtB, tet(G), floR, aadA2, dfrA12, aac(3)-Iia, aph(6)-Id, aph(3'')-Ib, sul2* |
|  | 12spc | - | CP092917.1 | *bla*_CTX-M-14_*, bla*_TEM-1B_*, rmtB, tet(G), floR, ant(3'')-Ia, dfrA12, aac(3)-Iia, aph(6)-Id, aph(3'')-Ib, sul2, bla*_KPC-2,_ *fosA6, bla*_SHV-178_ |
